# Treatment of bipolar depression with supraphysiologic doses of levothyroxine: a randomized, placebo-controlled study of comorbid anxiety symptoms

**DOI:** 10.1186/s40345-019-0155-y

**Published:** 2019-10-04

**Authors:** Maximilian Pilhatsch, Thomas J Stamm, Petra Stahl, Ute Lewitzka, Anne Berghöfer, Cathrin Sauer, Michael Gitlin, Mark A. Frye, Peter C. Whybrow, Michael Bauer

**Affiliations:** 1 Department of Psychiatry and Psychotherapy, Medical Faculty, Universitätsklinikum Carl Gustav Carus, Technische Universität Dresden, Fetscherstr. 74, 01307 Dresden, Germany; 2 Department of Psychiatry and Psychotherapy, Charité - Universitätsmedizin Berlin, Berlin, Germany; 3 Psychiatrische Universitätsklinik der Charité, St. Hedwig-Krankenhaus, Berlin, Germany; 4 Institute for Social Medicine, Epidemiology and Health Economics, Charité - Universitätsmedizin Berlin, Berlin, Germany; 5 Department of Psychiatry and Biobehavioral Sciences, Semel Institute for Neuroscience and Human Behavior University of California Los Angeles (UCLA), Los Angeles, CA USA; 6 Department of Psychiatry & Psychology, Mayo Clinic Depression Center, Mayo Clinic, Rochester, MN USA; 7 Department of Psychiatry, Psychotherapy and Psychosomatics, Brandenburg Medical School, Neuruppin, Germany

## Abstract

**Background:**

Symptoms of anxiety co-occur in a variety of disorders including in depressive episodes of bipolar disorder and in patients with thyrotoxicosis. Treatment of refractory bipolar disorder with supraphysiologic doses of levothyroxine (L-T4) has been shown to improve the phenotypic expression of the disorder and is associated with an increase of circulating thyroid hormones. However, it might be associated with somatic and mental adverse effects. Here we report the investigation of the influence of treatment with supraphysiologic doses of L-T4 on symptoms of anxiety in patients with refractory bipolar depression.

**Methods:**

Post-hoc analysis from a 6-week, multi-center, randomized, double-blind, placebo-controlled study of the effects of supraphysiologic L-T4 treatment on anxiety symptoms in bipolar depression. Anxiety symptoms were measured weekly with the Hamilton anxiety/somatization factor (HASF) score of the Hamilton Depression Rating Scale (HAMD) and the State- and Trait Anxiety Inventory (STAI).

**Results:**

Treatment of both groups was associated with a significant reduction in anxiety symptoms (p < 0.001) with no statistical difference between groups (LT-4: from 5.9 (SD = 2.0) at baseline to 3.7 (SD = 2.4) at study end; placebo: from 6.1 (SD = 2.4) at baseline to 4.4 (SD = 2.8) at study end; p = 0.717). Severity of anxiety at baseline did not show a statistically significant correlation to the antidepressive effect of treatment with supraphysiologic doses of L-T4 (p = 0.811). Gender did not show an influence on the reduction of anxiety symptoms (females: from 5.6 (SD = 1.7) at baseline to 3.5 (SD = 2.4) at study end; males: from 6.1 (SD = 2.3) at baseline to 4.0 (SD = 2.4) at study end; p = 0.877).

**Conclusions:**

This study failed to detect a difference in change of anxiety between bipolar depressed patients treated with supraphysiologic doses of L-T4 or placebo. Comorbid anxiety symptoms should not be considered a limitation for the administration of supraphysiologic doses of L-T4 refractory bipolar depressed patients.

*Trial registration* ClinicalTrials, ClinicalTrials.gov identifier: NCT01528839. Registered 2 June 2012—Retrospectively registered, https://clinicaltrials.gov/ct2/show/study/NCT01528839

## Background

Depression is the most difficult phase of bipolar disorder to treat (Bauer et al. 2018). Unfortunately, evidence from treatment trials in bipolar depression is sparse, and the role of antidepressants and their efficacy remains controversial (Gitlin 2018). Adjunctive treatment with thyroid hormones at supraphysiologic doses is one approach for treatment-refractory bipolar disorders for the acute depressive phase and maintenance treatment. While triiodothyronine (T3) demonstrated to be effective and safe in the treatment of affective disorders (Kelly and Lieberman 2009), the use of higher than normal, supplementary doses of levothyroxine (L-T4) has shown promise in several open-label studies, including for patients with rapid cycling (Bauer and Whybrow 1990), prophylaxis-resistance (Baumgartner et al. 1994; Bauer et al. 2002b), and with acute refractory uni- or bipolar depression (Bauer et al. 1998, 2002a, 2005). These beneficial findings have been confirmed more recently in placebo-controlled studies (Stamm et al. 2014; Bauer et al. 2016; Walshaw et al. 2018): in the multicenter placebo-controlled study of Stamm et al. (2014), supraphysiologic doses of L-T4 were added to continuing treatment with mood stabilizer and/or antidepressant medication in 62 patients with bipolar depression. After 4 weeks but not at the study end at week 6 HAMD scores in the L-T4 group were significantly lower compared to the placebo group. However, the secondary analysis of women (n = 32) revealed a significant difference between groups in mean change in HAMD score in favor of the L-T4 treated group. Patients from a subsample (N = 25) were enrolled to an imaging study assessing cerebral glucose metabolism with positron emission tomography (Bauer et al. 2016). Adjunctive treatment with supraphysiologic doses of L-T4 produced a significant decline in depression scores during the 6-week treatment, which was paralleled by restoration of metabolic activity in brain regions that are critically involved in the regulation of emotional processing and homeostasis. Recently, Walshaw et al. (2018) found that mood-stabilizing effects of adjunctive treatment with supraphysiologic doses of L-T4 were significantly higher compared to effects of T3 and placebo in 32 patients with treatment resistant rapid cycling.

Accordingly, treatment with supraphysiologic doses of L-T4 is recommended in several treatment guidelines for bipolar disorders (Grunze et al. 2010; Hirschfeld et al. 2010; Sachs et al. 2000; Yatham et al. 2018).

The hypothesis driving these studies was that increasing the availability of thyroid hormones to the brain changes the phenotypic expression of the disorder and is associated with improvement of mood that correlates with changes in brain metabolism in limbic areas (Bauer et al. 2005, 2016).

However, treatment with supraphysiological doses of thyroid hormones was erroneously classified as “thyrotoxic” and the patients treated as “hyperthyroid”, which limited prescription by psychiatrists, as Kelly (2015) and Pilhatsch et al. (2018) pointed out. Meanwhile, a large number of studies indicate good tolerability showing lack of signs and symptoms of thyrotoxicity in patients with affective disorders who receiving supraphysiologic doses of thyroid hormones (Bauer et al.^1998^, ^2005^; Stamm et al. 2014; Bauer et al. 2016; Kelly 2015; Kelly et al. 2016). Monitoring of symptoms typically associated with thyrotoxicosis demonstrated no evidence for specific side effects: several prospective observational studies found no evidence for bone loss (Gyulai et al. 2001; Ricken et al. 2012), disturbed sleep (Kraemer et al. 2011) or increased risk for cardiovascular intolerance (Pilhatsch et al. 2018) during treatment with high dose L-T4. Moreover, studies of supraphysiologic doses of thyroid hormones used to prevent the reoccurrence of thyroid cancer did also not show a risk for bone loss or cardiovascular sequela (Kelly 2015; Kelly et al. 2016).

Epidemiological studies have revealed high rates of comorbidity between bipolar and anxiety disorders (Grande et al. 2016). Symptoms of anxiety are common during depressive episodes in bipolar disorder (Bauer et al. 2010; Grande et al. 2016). Likewise, episodic anxiety, frequently in association with subjective awareness of tachycardia or arrhythmia, is also a common symptom in thyrotoxic patients (Bauer et al. 2013). Indeed, some of these diffuse anxious and dysphoric feelings have been reported in normal subjects when given high doses of levothyroxine (L-T4) (Bauer et al. 2002a). Hence, it has been argued that treatment with supraphysiologic doses of L-T4 may lead—similar to thyrotoxicosis—to an increase of anxiety symptoms in bipolar depressed patients. To date, no study has focused on anxiety as a psychological side effect during treatment of L-T4.

We therefore identified a need to disentangle specific effects on depressive and comorbid anxiety symptoms during treatment of bipolar depression with supraphysiologic doses of L-T4. Moreover, we wanted to investigate if treatment with supraphysiologic doses of L-T4 is tolerated worse by patients with anxious depression. To achieve this goal, we assessed trait and state anxiety during the randomized, placebo controlled double-blind trial of adjunctive therapy with supraphysiologic L-T4 for bipolar depression (Stamm et al. 2014), mentioned above. Specifically, we investigated (i) whether supraphysiologic doses of L-T4 would lead to an increase of anxiety symptoms; (ii) whether severity of anxiety symptoms at baseline would moderate the antidepressive effect of treatment with supraphysiologic doses of L-T4; (iii) whether a gender difference can be identified regarding the effect of supraphysiologic doses of L-T4 and placebo on anxiety symptoms.

## Methods

### Study design and sample

This 6-week, multi-center, randomized, double-blind, placebo-controlled study was previously described in detail (Stamm et al. 2014). Participants were 18–65 years of age and diagnosed with bipolar disorder according to the Diagnostic and Statistical Manual of Mental Disorders, Fourth Edition (DSM-IV), currently in a depressive phase. They had failed to respond to at least 6 weeks of treatment with a mood stabilizer and/or antidepressant (see below) at standard doses according to the WFSBP guideline (Bauer et al. 2007). Serum levels of lithium were within therapeutic ranges (0.5–0.8 mmol/L) for at least 2 weeks before enrollment. Exclusion criteria were: (1) an axis I disorder other than bipolar disorder, (2) ultra-rapid cycling, defined as ≥ 12 mood events in the previous year, (3) psychotic features, (4) a diagnosis of substance dependence or substance use (except for nicotine) over the previous year, (5) a clinically significant current medical illness, or (6) current or past thyroid disease or thyroid hormone treatment. Serum thyroid hormone [free thyroxine (fT4) and free triiodothyronine (fT3)] and thyroid-stimulating hormone (TSH) levels were required to fall within the normal reference range of the laboratory (fT4: 0.9–1.9 ng/dL; fT3: 2.6–5.1 ng/L; TSH 0.27–4.2 mU/L) in order to exclude subthreshold thyroid disease.

The study was carried out in accordance with the Declaration of Helsinki at six European centers. Ethical approval was obtained at each institution. All patients gave written informed consent. Please see Stamm et al. (2014) for a more comprehensive description of study design and procedures.

### Procedures and assessments

Participants with a baseline score of at least 14 on the 17-item Hamilton Depression Rating Scale (HAMD, Hamilton 1960) and a Young Mania Rating Scale (Young et al. 1978) score below 13, entered the double-blind study phase. After randomization, they were given either a supraphysiologic dose of levothyroxine (300 mcg/d) or placebo as an add-on to their stable (at least 6-week) pre-treatment medication with a prophylactic and/or antidepressive medication. Affective symptoms were also assessed with the Montgomery–Asberg Depression Rating Scale (MADRS; Montgomery and Asberg 1979). Of 74 patients enrolled into the study, 62 were randomized to the study groups (L-T4 vs. placebo).

Anxiety symptoms were operationalized using the Hamilton anxiety/somatization factor (HASF) score of the HAMD scale and the State- and Trait-Anxiety Inventory (STAI; Spielberger 1983). During the study, HAMD and STAI were assessed weekly from baseline until study end at week 6.

### Statistical analyses

Demographic and baseline clinical data were analyzed descriptively calculating means, standard deviations (SD), frequencies and contingency tables. Potential differences at baseline between the two treatment groups were examined using t-test and Chi^2^-test as appropriate. To assess the differences between groups concerning the outcomes we used t-tests, linear regression analysis as well as repeated-measures analysis-of-covariance (rmANCOVA) for analysis of the course of our outcomes over time. Outcome measures were HAMD, Hamilton anxiety/somatization factor (HASF) score, State-STAI and Trait-STAI. Independent variables were treatment (L-T4 vs. placebo), gender, and extent of comorbid anxiety symptoms at baseline (high vs. low). Age was included as covariate. Alpha error was adjusted to 5%. Mauchly’s Test of sphericity was used to test the assumption of sphericity. When sphericity was violated Huynh–Feldt corrections were performed. Missing follow-up data have been replaced by that subject’s previously observed value, i.e. the last observation was carried forward. Statistics were done using SPSS-22 for Windows.

## Results

### Demographical and clinical data

Baseline comparisons of both demographic characteristics and clinical parameters such as severity of depressive symptoms (HAMD) and severity of anxiety symptoms (STAI state and trait) revealed no significant differences between treatment groups (Table 1). Age tended to be higher in controls (p = 0.07) and was integrated as covariate in further analyses.

**Table 1:** Demographic and baseline clinical characteristics of participants with bipolar depression (intent-to-treat population)

Item	Levothyroxine (n = 31)	Placebo (n = 31)	Difference
Bipolar I/II, n	15/16	19/12	Chi^2^ = 1.042, df = 1, p = 0.307
Age, mean (SD), years	41.8 (12.8)	48.0 (14.2)	T = 1.8, df = 60, p = 0.07
Sex, male/female, n	14/17	16/15	Chi^2^ = 0.258, df = 1, p = 0.611
Total no. of mood episodes, mean (SD)	8.0 (7.8)	9.5 (9.2)	
Duration of illness (mean), years	12.3 (9.7)	18.1 (15.1)	T = 1.694, df = 52, p = 0.096
Mean HASF score (SD); n = 62	5.9 (2.0)	6.1 (2.4)	T = 0.351, df = 60, p = 0.727
Mean State-STAI score (SD); n = 61	58.2 (9.5)	57.4 (9.1)	T = − 0.347, df = 59, p = 0.727
Mean Trait-STAI score (SD); n = 61	58.4 (11.4)	57.9 (9.9)	T = 0.189, df = 59, p = 0.850
HAMD baseline score, mean (SD)	20.9 (3.0)	21.4 (4.3)	T = 0.549, df = 60, p = 0.585
MADRS baseline score, mean (SD)	28.8 (5.6)	30.3 (6.0)	T = 1.031, df = 60, p = 0.307

*HASF* Hamilton anxiety/somatization factor score, *STAI* State-Trait Anxiety Inventory, *HAMD* Hamilton Depression Rating Scale, *MADRS* Montgomery–Asberg Depression Rating Scale

Comparisons of thyroid measurements showed that serum fT4 levels of female participants were significantly elevated at baseline compared to males (0.9 ng/dL vs. 1.2 ng/dL; p = 0.01). As expected, treatment with L-T4 was associated with a significant increase of fT3 and fT4 and decrease of TSH compared to placebo at study end.

### Efficacy and tolerability outcomes

The primary efficacy outcome of the main study has been previously reported (Stamm et al. 2014). In summary, mean change in HAMD score was larger in the L-T4 group compared to the placebo group during the 6-week study period. At week 4, but not at the endpoint of the study, depressive symptoms of patients treated with L-T4 significantly improved. In a post hoc analysis, a gender effect was found: female participants treated with L-T4 demonstrated a significant reduction of HAMD scores compared to the placebo-treated group (Stamm et al. 2014).

With respect to tolerability no serious events occurred during the study. Regular assessments of ECG and body weight, measurements of blood pressure revealed normal results at baseline and indicated no significant changes during or after treatment. Monitoring of side effects typically associated with thyrotoxicosis with the “thyroid symptom list” (Bauer et al. 2002a) indicated no significant difference of such symptoms between groups at either the beginning or end of the study. However, three participants of the L-T4 group discontinued the study due to adverse events. In one of these cases mild thyrotoxicosis was diagnosed (Stamm et al. 2014).

### Anxiety measures

**Fig. 1:**
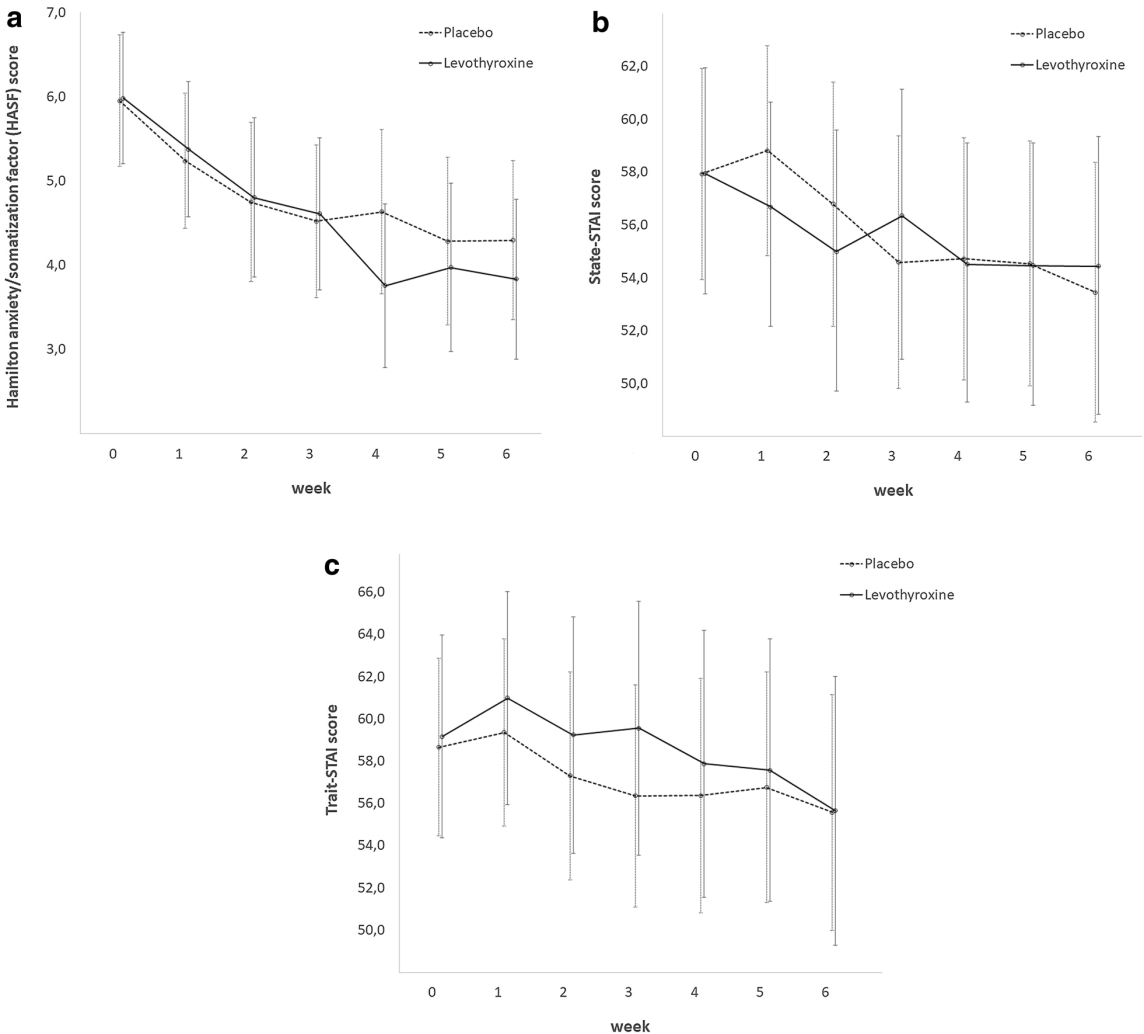
Course of anxiety symptoms during 6-week treatment of bipolar depression in both treatment groups (L-T4 vs placebo). **a** Change of Hamilton anxiety/somatization factor (HASF) score. **b** Change of State-STAI score. **c** Change of Trait-STAI score

We tested whether treatment with supraphysiologic doses of L-T4 leads to a change of comorbid anxiety symptoms compared to placebo. The mean HASF score at baseline was 5.9 (SD = 2.0, 95% CI [5.1;6.6]) for the L-T4 group and 6.1 (SD = 2.4, 95% CI [5.2;6.9]) for the placebo group (T = 0.351, df = 60, p = 0.727). As illustrated in Fig. 1a, treatment in this study was associated with a significant (31.7%) decrease of anxiety symptoms (HASF score) from 6.0 (SD = 2.2; 95% CI [5.4;6.5] at baseline to 4.1 (SD = 2.6; 95% CI [3.4; 4.7] after 6 weeks. (T = 5.4; p < 0.001). In the LT-4 group, HASF score decreased by 37.3% (from baseline to 3.7 (SD = 2.4; 95% CI [2.9;4.6]) at week 6. At study end there was no significant difference to the placebo group in which the HASF score decreased by 27.9% to 4.4 (SD = 2.8, 95% CI [3.4;5.4]). The analyses with the STAI measures led to similar results (Fig. 1b, c).

Repeated measures ANCOVA revealed no significant main effect of treatment (F (1, 59) = 0.132, p = 0.717) or time (F (4.19, 37.69) = 0.501, p = 0.744, Huynh–Feldt corrected). Age had no significant influence on the results. Interaction of time and treatment was also not significant (F (4.19, 37.69) = 0.826, p = 0.514, Huynh–Feldt corrected).

We further explored whether severity of anxiety symptoms at baseline would moderate the antidepressive effect of treatment with supraphysiologic doses of L-T4. Therefore, we examined the correlation between improvement of HAMD score from baseline to study end and the score for severity of anxiety at baseline, including HAMD baseline score as covariate. Linear regression analysis showed no statistical significance (F (2, 28) = 0.211, p = 0.811).

In the original publication, female patients benefitted more from treatment with supraphysiologic doses of L-T4 compared to male patients (Stamm et al. 2014). Therefore, we investigated whether a gender effect could be identified in the response of L-T4 treatment of anxiety symptoms. Seventeen female patients receiving L-T4 treatment had mean HASF scores of 5.6 (SD = 1.7, 95% CI [4.8;6.5])) at baseline indicating no significant difference to the 14 male participants (6.1; SD = 2.3, 95% CI [4.8;7.5]) (T = 0.688, df = 29, p = 0.497). At study end anxiety measures were reduced in both groups; the mean HASF score for female patients was 3.5 (SD = 2.4, 95% CI [2.3;4.8]) and for male patients 4.0 (SD = 2.4, 95% CI [2.6;5.4]). A repeated measures ANCOVA with gender as between subject factor revealed no main effect of time (F (4.5, 17.3) = 0.537, p = 0.730, Huynh–Feldt corrected) and no significant interaction of time and gender (F (4.5, 17.3) = 0.654, p = 0.644, Huynh–Feldt corrected). Gender as between-subject-factor had no significant influence on anxiety measures (F (1, 28) = 0.025, p = 0.877). These same analyses with State-STAI scores led to similar results (details not shown).

## Discussion

In this 6-week randomized, double-blind, placebo-controlled trial adjunctive treatment with supraphysiologic doses of L-T4 in bipolar depression was associated with a significant reduction in anxiety symptoms. Compared to placebo, L-T4 treatment led to a numerical reduction of anxiety symptoms. However, this study failed to detect a statistic significant difference in change of anxiety between bipolar depressed patients treated with supraphysiologic doses of L-T4 or placebo.

The anxious-depressive subgroup of participants showed at least as good a response as the non-anxious-depressive patients. No gender differences were identified with regard to anxiety reduction during L-T4 augmentation.

Because treatment with supraphysiologic doses of L-T4 leads to an increase of thyroid hormone levels and thyrotoxicosis is associated with psychosis and anxiety, findings from this study are of clinical interest.

Results emphasize that the sequelae of people with thyrotoxicosis are different than those in patients with mood disorders resulting from treatment with supraphysiologic doses of T3 or L-T4 (Bauer et al. 2002a; Kelly 2015; Pilhatsch et al. 2018) suggesting a common entity between anxiety symptoms and depressive symptoms in bipolar disorders. Additionally, previous positron emission tomography (PET) studies have shown that anxiety disorders are associated with increased glucose metabolism in limbic brain structures such as hippocampus, parahippocampus and amygdala (Bisaga et al. 1998; Sakai et al. 2005). Another PET study indicated that comorbid anxiety symptoms have specific regional cerebral metabolic correlates in the same core limbic areas of patients with bipolar depression (Bauer et al. 2010). Given that these patterns of altered metabolism have specifically been restored by supraphysiologic treatment with L-T4 in a subsample of patients from this study (Bauer et al. 2016), our results underline that L-T4 treatment is a valuable treatment option even in anxious depression from both a mechanistic and clinical perspective.

This study had several methodological limitations: it was a post hoc analysis; an observer-rated scale to measure anxiety specifically was not included (with the exemption of the HAMD scale containing anxiety items). Even if the sample appears relatively small, a post hoc power analysis on the repeated measures ANCOVA for the course of HASF score comparing LT-4 and placebo group shows a statistical power of 80%.

In conclusion, based on data from this study supraphysiologic doses of L-T4 do not appear to increase anxiety symptoms, indicating that when treating patients with supraphysiologic doses of L-T4 there appears no reason to withhold it in patients with high severity of anxiety symptoms at baseline. Therefore, comorbid anxiety symptoms should advocate and not limit considering treatment with supraphysiologic doses of L-T4 as a valuable option for treatment refractory patients with bipolar depression.

## Data Availability

The data will not be shared or made publicly available. Informed consent for this was not sought from the study participants prior to the collection of data.
